# High-content live cell imaging with RNA probes: advancements in high-throughput antimalarial drug discovery

**DOI:** 10.1186/1471-2121-10-45

**Published:** 2009-06-10

**Authors:** Serena Cervantes, Jacques Prudhomme, David Carter, Krishna G Gopi, Qian Li, Young-Tae Chang, Karine G Le Roch

**Affiliations:** 1 Cell, Molecular, and Developmental Biology Graduate Program, University of California, Riverside, CA, 92521, USA; 2 Department of Cell Biology and Neurosciences, University of California, Riverside, CA, 92521, USA; 3 Institute for Integrative Genome Biology, University of California, Riverside, CA, 92521, USA; 4 Department of Chemistry, & NUS MedChem Program of the Office of Life Sciences, National University of Singapore, 11754, Singapore; 5 Laboratory of Bioimaging Probe Development, Singapore Bioimaging Consortium, Agency for Science, Technology and Research (A*STAR), Biopolis, 138667, Singapore; 6 Department of Chemistry, New York University, New York, NY 10003, USA

## Abstract

**Background:**

Malaria, a major public health issue in developing nations, is responsible for more than one million deaths a year. The most lethal species, *Plasmodium falciparum*, causes up to 90% of fatalities. Drug resistant strains to common therapies have emerged worldwide and recent artemisinin-based combination therapy failures hasten the need for new antimalarial drugs. Discovering novel compounds to be used as antimalarials is expedited by the use of a high-throughput screen (HTS) to detect parasite growth and proliferation. Fluorescent dyes that bind to DNA have replaced expensive traditional radioisotope incorporation for HTS growth assays, but do not give additional information regarding the parasite stage affected by the drug and a better indication of the drug's mode of action. Live cell imaging with RNA dyes, which correlates with cell growth and proliferation, has been limited by the availability of successful commercial dyes.

**Results:**

After screening a library of newly synthesized stryrl dyes, we discovered three RNA binding dyes that provide morphological details of live parasites. Utilizing an inverted confocal imaging platform, live cell imaging of parasites increases parasite detection, improves the spatial and temporal resolution of the parasite under drug treatments, and can resolve morphological changes in individual cells.

**Conclusion:**

This simple one-step technique is suitable for automation in a microplate format for novel antimalarial compound HTS. We have developed a new *P. falciparum *RNA high-content imaging growth inhibition assay that is robust with time and energy efficiency.

## Background

Malaria continues to be a major public health issue in many parts of the developing world [1]. Each year 300 to 500 million new clinical cases are officially reported. In the mid-fifties, the World Health Organization (WHO) launched a worldwide malaria eradication campaign using effective and inexpensive therapeutics and insecticides in designated malaria-infected areas. The program resulted in the elimination of endemic malaria in developed countries and a significant reduction of cases in developing parts of the world. The emergence of chloroquine-resistant parasites and DDT-resistant mosquito vectors has led to a reappearance and spread of malaria in most of the developing world. With the absence of an efficient vaccine, worldwide resistance to all commonly used antimalarial drugs (quinine, aminoquinolines and antifolate derivates), and the concern of emerging resistance to our last defense against this disease (artemisinin-based combination therapies) [2], there is a dire need for new antimalarial strategies.

One approach to the discovery of new therapeutic agents involves the identification of inhibitory small molecules through whole parasite-based screens. In such assays, large collections of small molecule libraries can be tested against parasite growth in culture. For decades, accurate and reliable quantitative assessment of the drug effects on parasite growth has been achieved by blood smear microscopic examinations and *in vitro *measurement of parasitic uptake of radioactive substrates [3-5]. However, these methods are relatively expensive, require multi-step procedures and are time-consuming. They become impractical as technology advances and the volume of small molecule libraries increases. Over the past few years, a number of new techniques have been developed to improve the cost and compatibility for today's automated high-throughput screening (HTS) facilities. These techniques include colorimetric assays [6]; such as, fluorescence-based assays that measure parasite nucleic acids using fluorescent dyes (e.g. Hoechst [7,8], PicoGreen^® ^[9], SYBR Green I [10,11], YOYO-1 [12] and DAPI [13]), or stable expression of chimeric fluorescent protein [14]. All these techniques have been used and adapted in automated or semi-automated HTS analyses using microplate readers or flow cytometry, and have proven to be reliable and cost-effective [12,13,15,16]. However, while these techniques allow the quantification of parasite growth in human erythrocyte cultures, they detect an average response from the whole population and are inapt to efficiently detect the drug effect at the morphological level. Morphological analysis can give additional information regarding the parasite stage affected by the drug and eventually an indication of the drug's mode of action. For such analyses, investigators are still compelled to examine Giemsa-stained infected blood smears under brightfield microscopy [15-17]. While today this methodology can be completed using a semi-automatic image analysis system [18], it requires a laborious multi-step preparation of fixed, stained blood smears and an experienced technician.

Recently, the development of high-throughput cellular imaging has emerged as a crucial tool to allow the integration of biologically complex effects into drug discovery. Such techniques can overcome the limitations of cellular-based high-throughput screening that measure a "simple" survival count by detecting morphological changes of individual cells in a microplate well. Cellular imaging technologies have been used in all stages of drug discovery including target discovery or mode-of-action studies [19]. When confocal microscopes and image-analysis tools are combined, cellular imaging platforms can facilitate the detection of cytotoxicity or cellular phenotypic changes in a cell population, and lead to the discovery of new drug targets [20]. Membrane permeable fluorescent molecules have been one of the most viable tools for live cell imaging technology [13]. Several permeable dyes, such as nucleic acid dyes (e.g. Hoechst), are commercially available and have been used in malaria high-throughput screening. While DNA measurement has been shown to be efficient in detecting parasite growth in the high-throughput screening format, the use of such dyes for parasite structural analysis is limited. Specific RNA quantification, which also correlates with cell growth and proliferation, could significantly increase parasite detection and improve the spatial and temporal resolution of the malaria parasite under drug treatment. However, cell-permeable RNA specific dyes are not readily available and are limited in their successful use, even though the exploitation of both permeate DNA and RNA dyes would be particularly valuable for high-throughput screening and high-content imaging in *Plasmodium*.

To identify malaria parasite specific RNA probes that could improve live cell microscopy imaging, we have screened a combinatorial library of 125 fluorescence styryl molecules [21,22] with infected red blood cells in a microplate format. Microplates were analyzed using the BD Pathway HT, an automated confocal imaging workstation. This inverted confocal microscope with temperature and carbon dioxide regulation chamber allows suspension cultures to be viewed without a fixation step. Fourteen dyes were found to have fluorescence intensity comparable or superior to DAPI staining. Out of the fourteen dyes, three dyes displayed a higher affinity to RNA. They exhibit a specific RNA staining pattern representative of the different morphological stages of the malaria parasite erythrocytic cycle. Using the identified RNA dyes and a high-throughput confocal imaging system, we have developed a sensitive and simple one-step fluorescence-based assay for use in *Plasmodium *high-content imaging. Inhibitory concentrations of known antimalarials using our image-based assay were similar to current nucleic acid dye and spectrophotometer assays. This new technology is expected to enhance the malaria drug discovery program and could eventually be adapted as an automated solution to screen parasitemia in infected patients.

## Methods

### *P. falciparum *Culture

3D7 and Dd2 *P. falciparum *malaria parasites (MRA-102, 156, MR4, ATCC^® ^Manassas, Virginia) were cultured in human type O+ erythrocytes in complete medium (RPMI 1640, 10 mg/ml Gentamicin (Gibco), 1.36 g/l Hypoxanthine (Acros), 1 M HEPES (Sigma), 7.5% Sodium Bicarbonate (Gibco), 20% Glucose (MP Biomedical), 1 M NaOH (Sigma), 20% Albumax (Gibco), 5% human serum) as previously described [23]. Cultures were maintained in 25-cm^2 ^flasks (Corning) at a volume of 10 ml and were gassed for 30 s with an environment of 3% CO_2_, 1% O_2_, and 96% N_2_, then incubated at 37°C. Synchronization of culture was achieved through sorbitol lysis of mature stage using 5% sorbitol (Fisher) fine-tuned by another lysis 8 hours later [24].

### Dye Preparation

Microwell plates already containing a library of RNA-probes were diluted in DMSO to 10 mM. A screening of 125 different RNA-probes were performed by diluting infected erythrocytes to 0.025% hematrocrit with a 6% parasitemia into optical bottom 96-well assay plates (Costar #3614, Corning, NY) containing 240 μl of complete medium. RNA-probes were added to a final concentration of 1 μM, 5 μM, 7.5 μM, and 10 μM with a total DMSO level of 0.5%. Plates were incubated in the dark for 30 minutes at 37°C. During microscopic analysis, the BD Pathway HT (BD Biosciences Bioimaging, Rockville, MD) temperature was regulated at 37°C. Each well was fluorescently imaged using fluorescence combinations of Semrock (Rochester, NY) DAPI, CFP, GFP, YFP and Texas Red BrightLine filter sets. We found that 5 μM was the optimal working concentration.

### RNA versus DNA specificity solution assay

DNA was extracted from *P. falciparum *using an adapted phenol/chloroform extraction. Infected erythrocytes were harvested and brought to a 50% hematocrit in PBS. Samples were incubated with cell lysis solution (Promega #A7933, Madison, WI) for 10 minutes at room temperature, followed by centrifugation. After removing the supernatant, pellet was incubated at 55°C for an hour in lysis buffer containing 4 M guanidine HCl (Promega), 10% SDS (Promega), and 20 mg/ml Proteinase K (New England Biolabs), and left overnight at 4°C. DNA was then extracted using Phenol/chloroform/isoamyl alcohol (Sigma) following the standard procedures.

RNA was extracted from infected erythrocytes with Trizol LS (Life Technology), as previously described in Le Roch *et. al*. 2003 [23].

To scan fluorescence emission, the SpectraMAX GeminiEM (MDS Analytical Technologies, Toronto, Canada) and the SoftMax Pro program were used. Using black, clear bottom, 96-well assay plates (Costar #3904), 10 two-fold serial dilutions were performed with extracted DNA and RNA starting at 200 μg/ml. Steady state concentrations of RNA or DNA at 10 μg/ml, were mixed with two-fold serial dilutions of the opposing nucleic acid. 132A, 107E, and 107F RNA probes were added to wells at a 10 μM concentration. Endpoint readings with an excitation of 500 nm, cutoff at 515 nm and emission of 605 nm for 132A, and excitation of 435 nm, cutoff at 605 nm and emission of 610 nm for 107E and 107F. DNA and RNA concentration as a function of fluorescence intensity was plotted with SigmaPlot.

### Counter Stain

Synchronized infected erythrocytes were stained with nuclear dye DAPI (Molecular Probes #D21490, Eugene, OR) or Hoechst 34580 (Molecular Probes #H21486, Eugene, OR) diluted in H_2_O and added to reach a final concentration of 20 ng/μl or 5 μg/ml respectively. RNA specific dyes were then added to reach a concentration of 5 μM and plates were incubated with both dyes in the dark at 37°C for 30 minutes. Images were taken using the BD Pathway HT with the temperature regulated at 37°C. A macro, an instruction set for the Pathway HT, was designed to take images of the same frame using the DAPI filter wheel, then the corresponding filter for RNA dye, then transmitted light, auto focusing for each image, moving to another frame in the same well, and to repeat in subsequent wells.

### Relative Fluorescence

Synchronized infected erythrocytes were diluted with uninfected erythrocytes in complete media (i.e. 0%, 1.5%, 3%, 4.5%, 6%). Diluted probes were added to reach a final concentration of 5 μM, incubated in the dark for 30 min at 37°C and the BD Pathway HT measured fluorescence intensity. Data analyses were performed using Microsoft Excel 2004 for Mac. Data points were graphed by 1) calculation of the mean of triplicates per sample condition, 2) subtraction of the fluorescence background, and 3) conversion to relative fluorescence units.

### Quantitative assay evaluation

Three 96-well microplates with parasite-infected erythrocytes were used as a positive control, and three microplates with uninfected erythrocytes were used as a negative control. All plates were diluted to a 0.025% hematocrit, and 6% parasitemia. 107E, 107F, and 132A dyes were added to microplates and the Pathway HT determined fluorescence intensity. The formula z' = 1 - (3SD_+ _+ 3SD_-_)/|Ave_+ _- Ave_-_| was used to caculate the Z' value. Where SD_+ _represents the positive control standard deviation, SD_- _the negative control standard deviation, Ave_+ _the mean value of the positive control, and Ave_- _the mean value of the negative control.

### IC_50_

3D7 parasite cultures were incubated with 0.1 nM, 0.4 nM, 1.2 nM, 3.7 nM, 11.1 nM, 33.3 nM, 100 nM, and 500 nM concentrations of chloroquine or artemisinin (Sigma #C6628-25G, St. Louis, MO) for 72 hours. Dd2 parasite cultures were incubated with 5 nM, 25 nM, 75 nM, 100 nM, 150 nM, 200 nM, 300 nM, and 500 nM concentrations of chloroquine. RNA dyes were added to reach 5 μM final concentration and incubated in the dark for 30 minutes at 37°C. Microscopic analysis was performed at a regulated temperature of 37°C. Montage images of 4 by 4 frames were acquired with transmitted light and the corresponding filter for RNA dyes. Images were merged to verify infected erythrocytes by having the fluorescent target overlap with visible hemazoin in transmitted light images. The Pathway HT calculated the parasitemia by using the Region of Interest (ROI) function to count fluorescent parasites. Parasitemia was determined by manual counts of fluorescent images and Giemsa-stained blood smears, and compared to Pathway HT counts. Samples of identical cultures were taken to perform a SYBR Green assay as previously described [16]. Data were analyzed using Microsoft Excel for Mac, and graphs were plotted using SigmaPlot 10 (Systat).

## Results

### Screening of styryl dye library and identification of three RNA dyes

**Figure 1:**
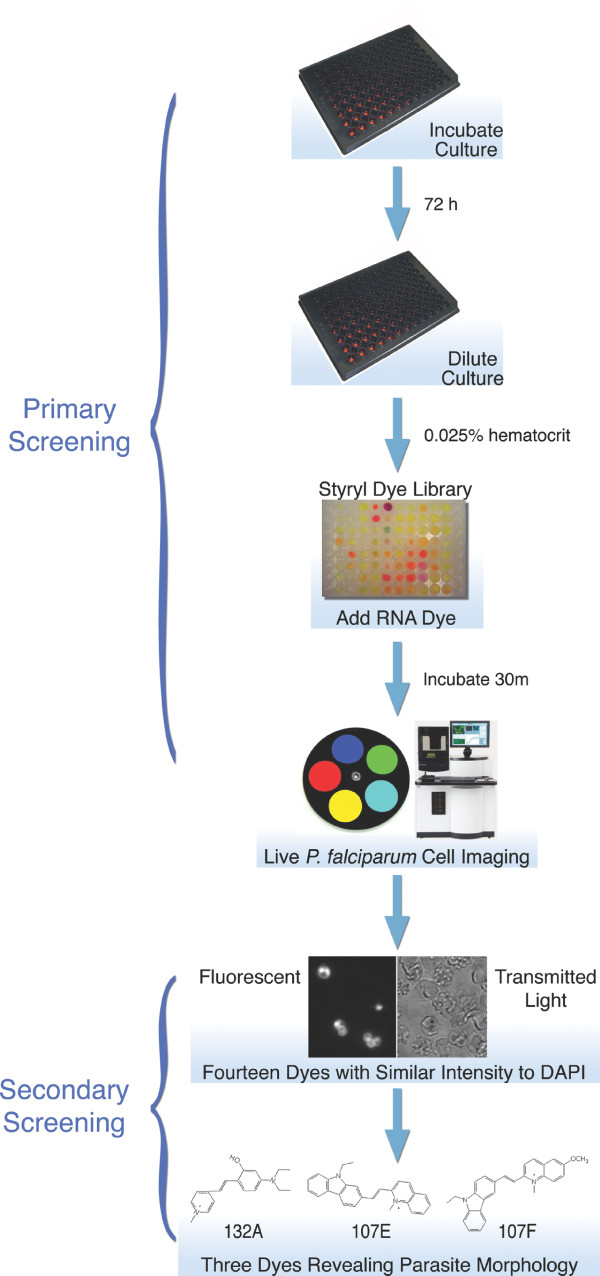
**Design of the RNA imaging P. falciparum growth assay**. Cultures were incubated in a 96-well microplate with antimalarial compounds in complete medium for 72 hrs under a standard gas environment at 37°C. After the drug incubation period, cultures were diluted to a 0.025% hematrocrit to form a monolayer of erythrocytes in a 96-well microplate. RNA dyes were added and incubated at 37°C for 30 min to stain parasites in infected erythrocytes. An automated fluorescent microscope, the Pathway HT, was used to detect and obtain images of fluorescent stained parasites, as described in Materials and Methods. Transmitted light images were also obtained to verify erythrocyte infection. Fourteen dyes were found to have similar intensity to DAPI and SYBR Green I in the primary screen. 132A, 107E, and 107F dyes were found to have a higher affinity to RNA, displaying a diffused staining throughout the parasite, in the secondary screening.

To identify new parasite RNA dyes, we screened a library of styryl compounds. Styryl dyes were synthesized and purified using a condensation reaction between methylated-pyridine derivatives and aldehydes, as previously described [21,22]. Cultures were diluted in microplates and incubated with the styryl compounds for 30 minutes at 37°C in the dark. Live cell imaging was performed using the Pathway HT microscope with temperature regulated at 37°C. This automated spinning disk confocal microscope with a motionless stage allows a monolayer of erythrocytes to be observed without fixation and minimal sample disruption. The Pathway HT was programmed to auto-focus to acquire transmitted light and fluorescent images in a special optics 96-well microplate format. Our Pathway HT is customized with high efficiency Semrock bandpass filters, and samples were imaged with all fluorescence combinations of Semrock DAPI, GFP, FRET, CFP, YFP, and Texas Red BrightLine filter sets to measure fluorescence intensity of the initial 125 RNA probes screened (figure 1). From the initial 125 stryrl dyes, fourteen RNA-probes were found to have fluorescent intensities similar or superior to 4'-6-diamidino-2-phenylindole (DAPI) and SYBR Green DNA staining.

**Figure 2:**
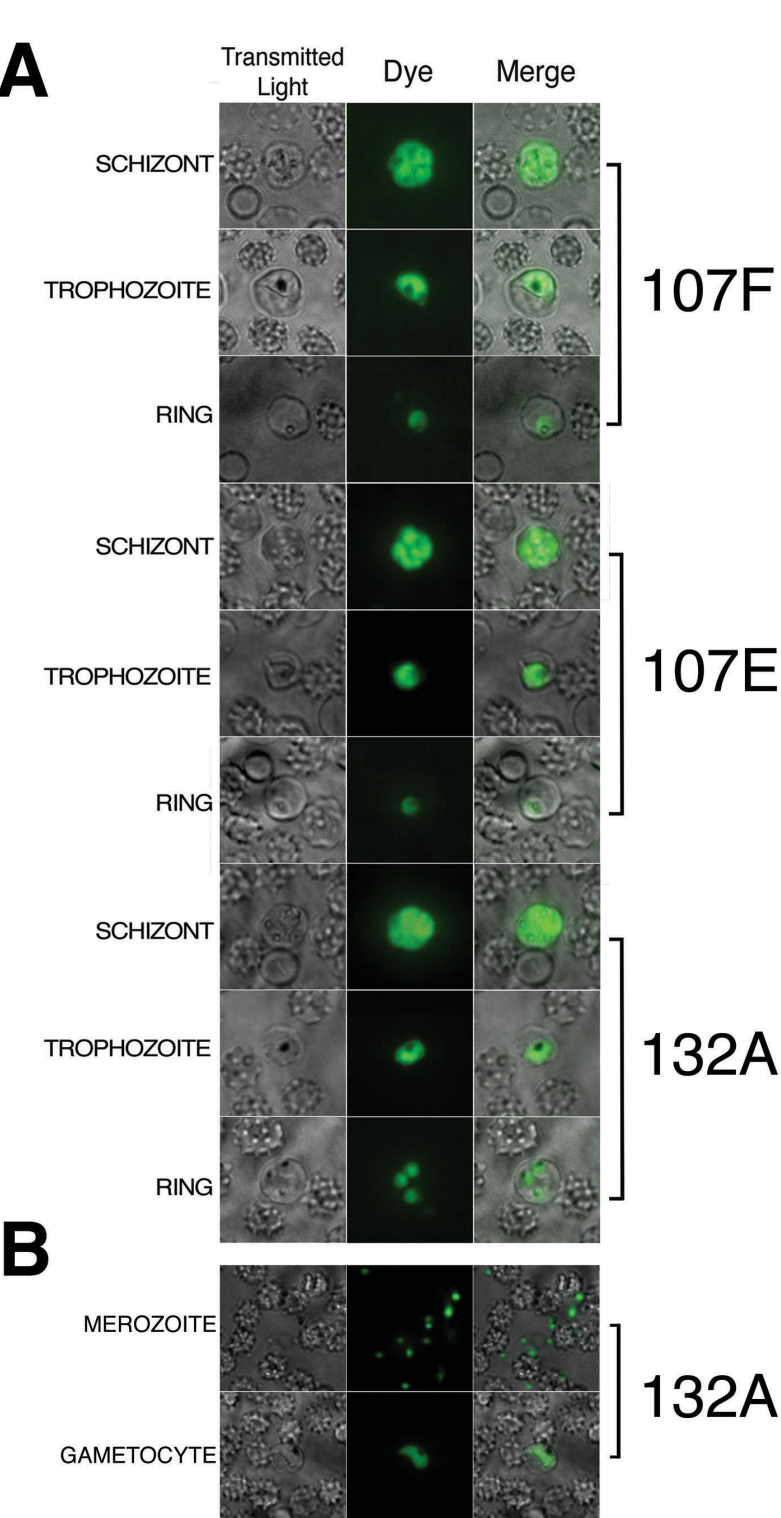
**Morphological analyses**. A) RNA dye staining of *P. falciparum *during schizont, trophozoite, and ring stages of erythrocyte life cycle were colored in green. RNA dyes were obtained using excitation 438/24 and emission 542/27 for 107F and 107E, and a red filter excitation 500/24 and emission 624/40 for 132A. B) RNA dyes are sensitive enough to detect merozoites and gametocytes in culture.

Out of the 14 dyes selected, three exhibited a higher affinity to RNA and displayed characteristic nucleolus targeting with a diffused staining pattern throughout the cytoplasm in all intraerythrocytic stages observed. Fluorescence properties of RNA-probes 132A and 107E were analyzed at the reported excitation 500 and emission 610 [21]. We found the YFP 500/24 excitation filter and Texas Red 624/40 emission filter gave the best fluorescent signal with dye 132A; while the long Stokes shift CFP 438/24 excitation and YFP 542/27 emission filter combination worked best for 107E and 107F (figure 1). To determine the optimum working dyes concentration 10 μM, 7.5 μM, 5 μM, and 2.5 μM were tested in infected erythrocytes. Fluorescent images taken with a 5 μM concentration provided an optimum signal to noise ratio. The three dyes demonstrated a signal to noise ratio of 14:1 for 107E, 11:1 for 107F, and 16:1 for 132A. The signal to noise ratio of 132A supports previous findings of the coupound's fluorescent emission fold range [21]. Little photobleaching and no visible change in cell morphology due to toxicity were detected during the analyses (figure 2A). The selected RNA dyes were sensitive enough to identify small-sized parasites, merozoites, and provided detailed images to differentiate between asexual, trophozoite, and early sexual stages of the malaria parasite (figure 2B).

**Figure 3:**
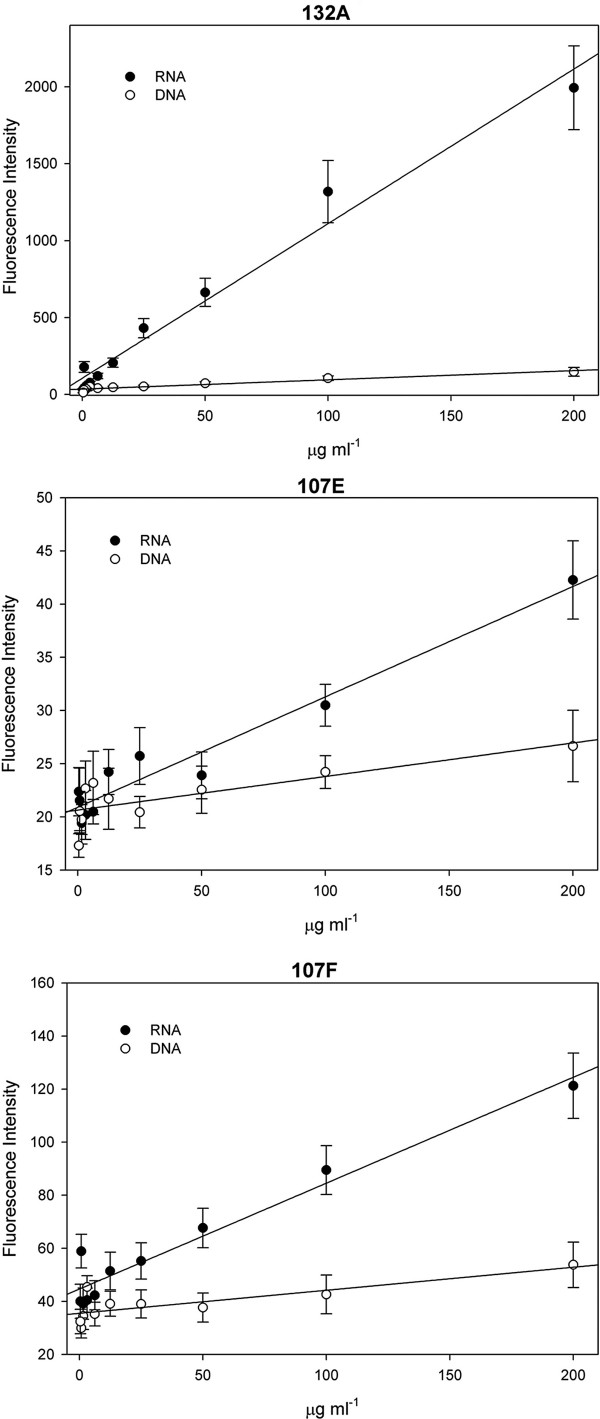
**RNA versus DNA specificity solution assay**. Fluorescence intensity of two-fold serial dilutions of *P. falciparum *RNA and DNA, with a 10 μM concentration of RNA probes. Dyes display a higher fluorescence intensity with RNA than DNA.

To determine if the RNA probes specifically target RNA, an RNA versus DNA specificity solution assay was performed. 132A, 107E, and 107F at a 10 μM concentration were added to two-fold serial dilutions of *P. falciparum *RNA and DNA. Fluorescence intensity as a function of RNA and DNA concentrations, displayed a higher response when bound to RNA when compared to DNA (figure 3). These results correspond to better nucleoli targeting in the live cell [21]. Furthermore, 132A exhibited a higher response in fluorescence intensity when bound to RNA in a sample containing DNA [see additional file 1].

**Figure 4:**
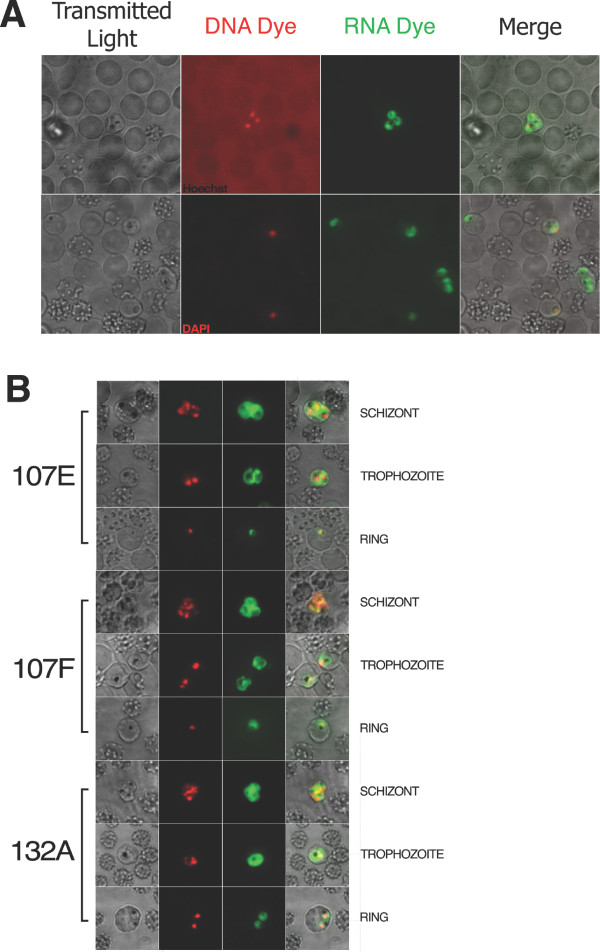
**Counter-staining analyses using fluorometric DNA and RNA dyes**. A) Counter-staining with DNA dyes demonstrates the sensitivity, permeability and the adequate signal to noise ratio of RNA dyes. Hoechst staining has a significant amount of background compared to 132A. 132A detected parasites that were overlooked by DAPI staining. B) Counter staining with nucleic acid dye DAPI, in red, and RNA dyes 107F, 107E, and 132A, in green, at schizont, trophozoite, and ring stages of erythrocytic cycle. Images obtained by the Pathway HT were grayscale; red and green colors were artificially added to emphasize localization.

To compare selectivity and sensitivity of our selected dyes with classical DNA staining, cell cultures were counterstained with the nucleic acid stain DAPI and Hoechst. Dyes 107E, 107F, and 132A efficiently permeated infected erythrocytes without the aid of lysis buffers or detergents. 132A displayed greater permeable properties and sensitivity in detecting parasites than DAPI (figure 4A), which is used in current HT antimalarial screens. High-content imaging can benefit by using both DNA and RNA dyes to assist in parasite stage analyses. Synchronized cell cultures stained with 107E, 107F, and 132A RNA dyes, and the DNA-specific dye DAPI, allowed the detection of all parasite asexual stages (figure 4B). Co-localization was observed in the nucleus, but RNA dyes targeted the nucleolus and diffused throughout the parasite allowing the visualization of parasite morphology at every erythrocytic stage of the malaria parasite. These data demonstrate the capability of RNA dyes to permeate parasite membranes more efficiently than DAPI staining and significantly increases the quality of the parasite phenotypic analysis.

### Relationship between parasitemia and fluorescence intensity and validation of RNA dyes for their use in a high-throughput malaria growth assay

**Figure 5:**
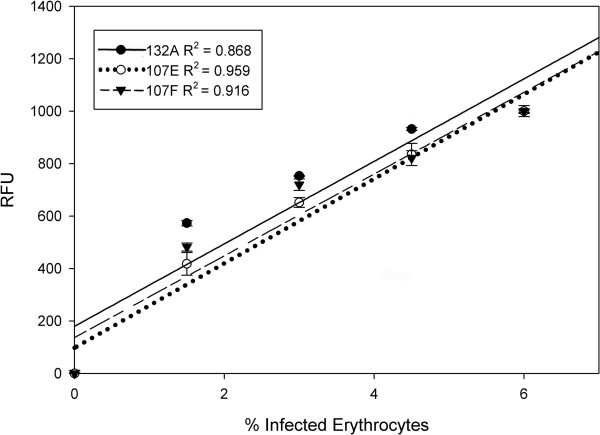
**Evaluation of the percentage of P. falciparum-infected erythrocytes using fluorescence intensity obtained by the Pathway HT**. Serial dilution of synchronized infected cultures (6% parasitemia) of *P. falciparum *3D7 strain were conducted as described in Materials and Methods. A linear relationship was observed between RFU and percentage of infected erythrocytes. Error bars represent the standard deviation of the mean from three independent samples.

To determine the relationship between infected erythrocytes and relative fluorescence intensity when stained with RNA dye, we compared the fluorescence intensity to the percentage of parasite-infected red blood cells. Serial dilutions of infected erythrocytes with 5 μM of RNA dye were prepared in triplicate and analyzed using the Pathway HT. As shown in Figure 5, an almost linear relationship between the percentages of infected red blood cells and the fluorescence signal was observed with an *R*^2 ^value of 0.868, 0.959 and 0.916 for 132A, 107E and 107F, respectively. To further validate our assay, we calculated the Z-factor values for each of the selected dyes. The Z-factor is a statistical characteristic for HTS assays used to compare and evaluate the quality of high-throughput assays [25]. A Z-factor value equal to one is an ideal assay, while values between 1 and 0.5 are considered excellent. The Z-factor was determined for each of our three selected dyes in a 96-well microplate and exhibited 0.689, 0.706, and 0.728 Z'- values for 107E, 107F, and 132A, respectively. These data demonstrate the ability to determine parasitemia based on relative fluorescence units (RFU), and validates the use of RNA probes for high-throughput live cell imaging.

**Figure 6:**
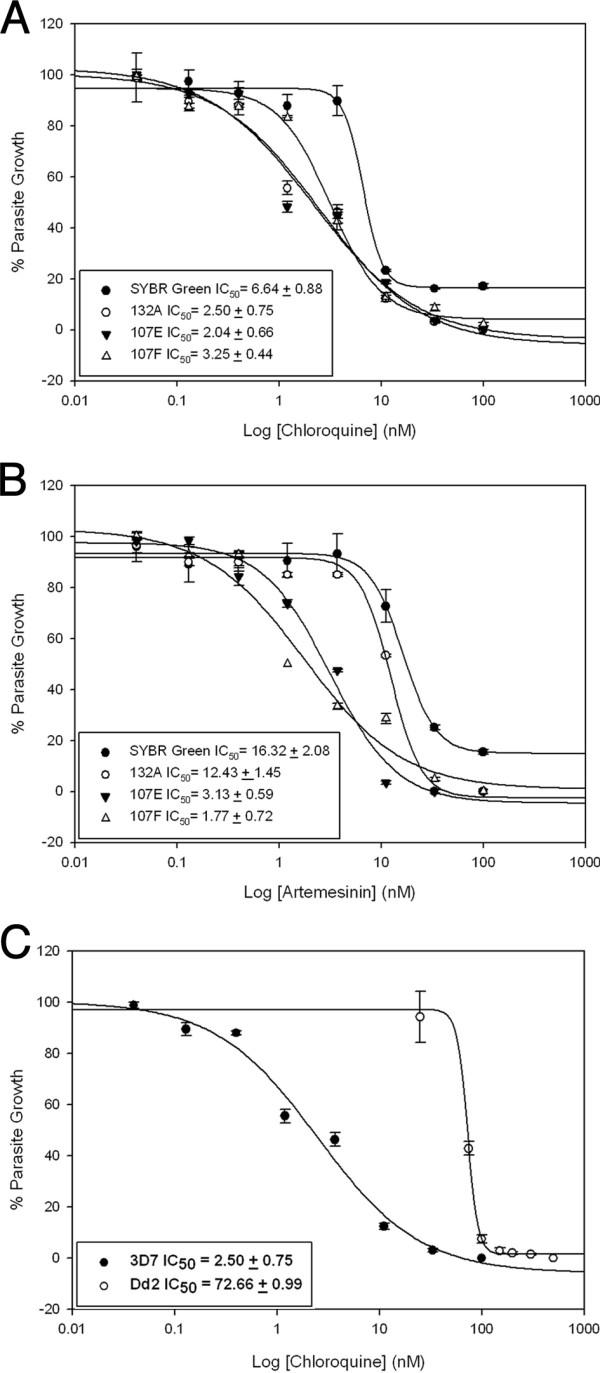
**Validation of parasite growth inhibition measurement by known antimalarials**. IC_50 _values for 3D7 parasite cultures treated with chloroquine (A) and artemesinin (B) were calculated using images obtained by the Pathway HT and compared to the classical SYBR Green assay. C) IC_50 _values determined with our high-content assay using fluorometric RNA dye 132A for parasite chloroquine sensitive 3D7 and chloroquine resistant Dd2 strains.

To ensure that the activities of antimalarials measured by RNA dye and image analysis were consistent with established parasite growth inhibition assays, we measured the IC_50 _values of known antimalarials (chloroquine and artemisinin), and of chloroquine sensitive and resistant parasite strains using the Pathway HT. The Pathway allows the detection of fluorescent parasites based on set parameters and numbers them according to size and intensity. Furthermore, the Pathway HT montage function compiles multiple frames into one tiled image that provides a large area of observation [see additional file 2]. These data can then be used to determine percentage of parasite growth compared to drug dose. IC_50 _values obtained using RNA dye high-throughput confocal microscopy assay were comparable to IC_50_values measured by the widely used SYBR Green assay (Figure 6). Data were further validated by microscopic analyses of Giemsa-stained blood smears (data not shown). While the IC_50 _values obtained with the three selected dyes are closely related to each other, they did not produce identical results. This can be explained by the differences observed in signal to noise ratios and the small disparate Z'-values obtained for each dye. To further validate the sensitivity of our assay, we calculated the minimum parasitemia detected by the Pathway HT above background. The Pathway HT detected 1 single parasite out of 12,000 RBCs, corresponding to a 0.0083% parasitemia, confirming the sensitivity of the high-content imaging assay.

**Figure 7:**
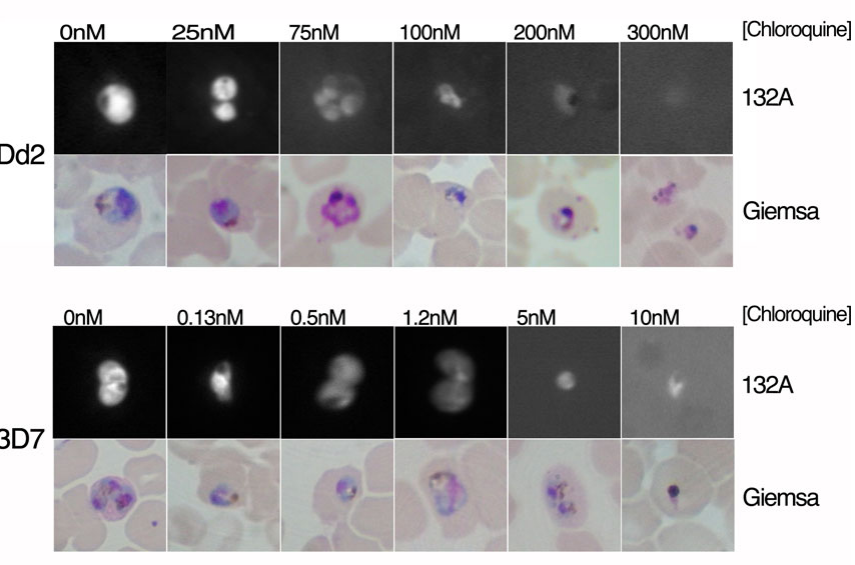
**Morphological analysis of 3D7 and Dd2 parasites using the Pathway HT**. Parasite synchronized cultures were incubated for 72 hours with various concentrations of chloroquine, live-stained with the fluorometric RNA dye 132A and fixed-stained with Giemsa. Detailed images of infected erythrocytes show a decrease in fluorescence intensity, loss of nucleolus staining, and change of parasite morphology with increasing drug concentration.

To confirm that the developed high-content live cell imaging assay can combine IC_50 _values with morphological examination of the parasite under drug treatment, images obtained with parasite Dd2 and 3D7 strains were compared to Giemsa-stained blood smears (Figure 7). Morphological phenotypes of parasites observed between the two techniques were highly similar. Using the fluorometric RNA staining dye 132A, parasites display a loss of nucleolus staining, and a decrease in signal intensity with an increase in drug concentration. All together, these data demonstrate that RNA dyes are sufficiently sensitive and reproducible to identify the inhibition effects of antimalarial compounds in a HTS screening and high-content imaging format.

## Discussion

In the past few years, cellular imaging has emerged as a critical tool to efficiently integrate the cellular complexity into drug discovery. Continuous technical improvements in fluorescence, confocal microscopy platforms, and image analysis have enhanced the detection and improved the resolution by which an individual molecule can act in a cell. High-content cellular imaging screenings recently lead to the discovery of small molecule inhibitors of novel cellular targets in human cancer cell lines [20]. Technological progress observed in cellular imaging toward human diseases needs to be adapted to the malaria parasite to enhance its drug discovery program. It is essential to integrate a spatial and temporal phenotypic analysis of the parasite under drug pressure in order to identify small molecule inhibitors that block major specific parasitic events (e.g. parasite invasion, division or egress). These types of phenotypic studies will improve the biological relevance of malaria HTS.

To address this need, we screened a combinatorial library of fluorescent styryl molecules, which contain RNA probing compounds that have been successfully identified in human cell lines [21,22]. Using a confocal microscope platform, the Pathway HT, we identified three RNA dyes with strong imaging properties relative to commercially available DNA dyes in live parasites. The selected dyes, 107E, 107F, and 132A are membrane permeable, diffuse throughout the cytoplasm to display parasite morphology, target nucleoli, and can be used in conjunction with DAPI staining. Out of the three dyes 132A exhibited the best signal to noise ratio and Z'-value, therefore this dye appears to have the best properties and will be used in future assays. Such characteristics allow the observation of RNA quantity and distribution in relation to the organization of the DNA within the parasite. Simultaneous DNA and RNA staining significantly increases the cell structural organization and facilitates the morphological analysis of *Plasmodium *in culture. While GFP fluorescence proteins have been exploited as tools to facilitate the dynamic behaviors of proteins and parasite phenotype examination in a real-time manner, these techniques require the use of modified cell lines, which are not the optimal choice for a large, small molecule screen against different *Plasmodium *drug resistant and sensitive isolates. In addition, optimum DNA staining, which has been successful in malaria HTS, requires the addition of lysis buffer and/or a fixation step that prevents the observation of live parasites and increases the screen's complexity. Although it is possible using DAPI staining to see numerous nuclei in close enough proximity to identify schizont stages, the differentiation between other stages of the erythrocyte life cycle is more challenging. The use of RNA dye together with DNA staining provides a dynamic morphology of all parasite erythrocytic cycle stages. It gives accurate information on parasite RNA content and its transcriptional activity, as well as information on cytotoxicity effects on erythrocytes and parasites.

## Conclusion

In conclusion, our IC_50 _results clearly demonstrate the feasibility of RNA directed fluorescence-based assays for high-throughput screening of antimalarials. Furthermore, these type of HTS and high-content imaging assay analyses can be defined and programmed automatically on the Pathway HT, avoiding the need of an experienced or biased microscopist to analyze the images. This new *Plasmodium *RNA dye growth inhibition and high-content imaging assay is a robust, cost effective, fast, and simple one-step technique suitable for automation in 96- to 384-well plates. It will be an ideal technique for a secondary screen validation, help to determine the stage specificity of drugs and potentially indicate a drug's mode of action. When combined with a similar mammalian screen, we should be able to identify the parasite specific compounds. It is also important to highlight that the sensitivity of such technique should allow the method to be adapted for the identification of infected blood samples of patients.

## Abbreviations

**HTS**: high-throughput screen; **RFU**: relative fluorescence units.

## Authors' contributions

YTC and KLR conceived the study. SC carried out screening and microscopic analyses. JP and SC prepared samples and collected spectrometer data. DC participated in the assay design. QL synthesized original library of probes, and GGK synthesized selected compounds. SC, JP, YTC and KLR contributed analyzing experimental studies. KLR drafted the manuscript. All authors read, edited and approved the final manuscript.

## Supplementary Material

Additional file 1 **DNA: RNA binding assay**. Graph represents 132A staining of RNA and DNA in a mixed population, with one nucleic acid kept at a steady state and the other serially diluted, where the maximum fluorescence intensity was set to one. Steady state RNA was observed to have a higher fluorescence intensity than serially diluted RNA, indicating 132A has a higher fluorescent signal when bound to RNA. Click here for file

Additional file 2 **Merged montage images**. A tiling of two by two images of transmitted light and fluorescent parasites, were taken with the Pathway HT. Decreased parasitemia with increasing concentration of chloroquine was observed with the 3D7 strain. Click here for file
