# Supplementary figures and images for: High-content live cell imaging with RNA probes: advancements in high-throughput antimalarial drug discovery
Source: BMC Cell Biol. 2009 Jun 10;10:45. doi: 10.1186/1471-2121-10-45 (PMC2702272; doi:10.1186/1471-2121-10-45)

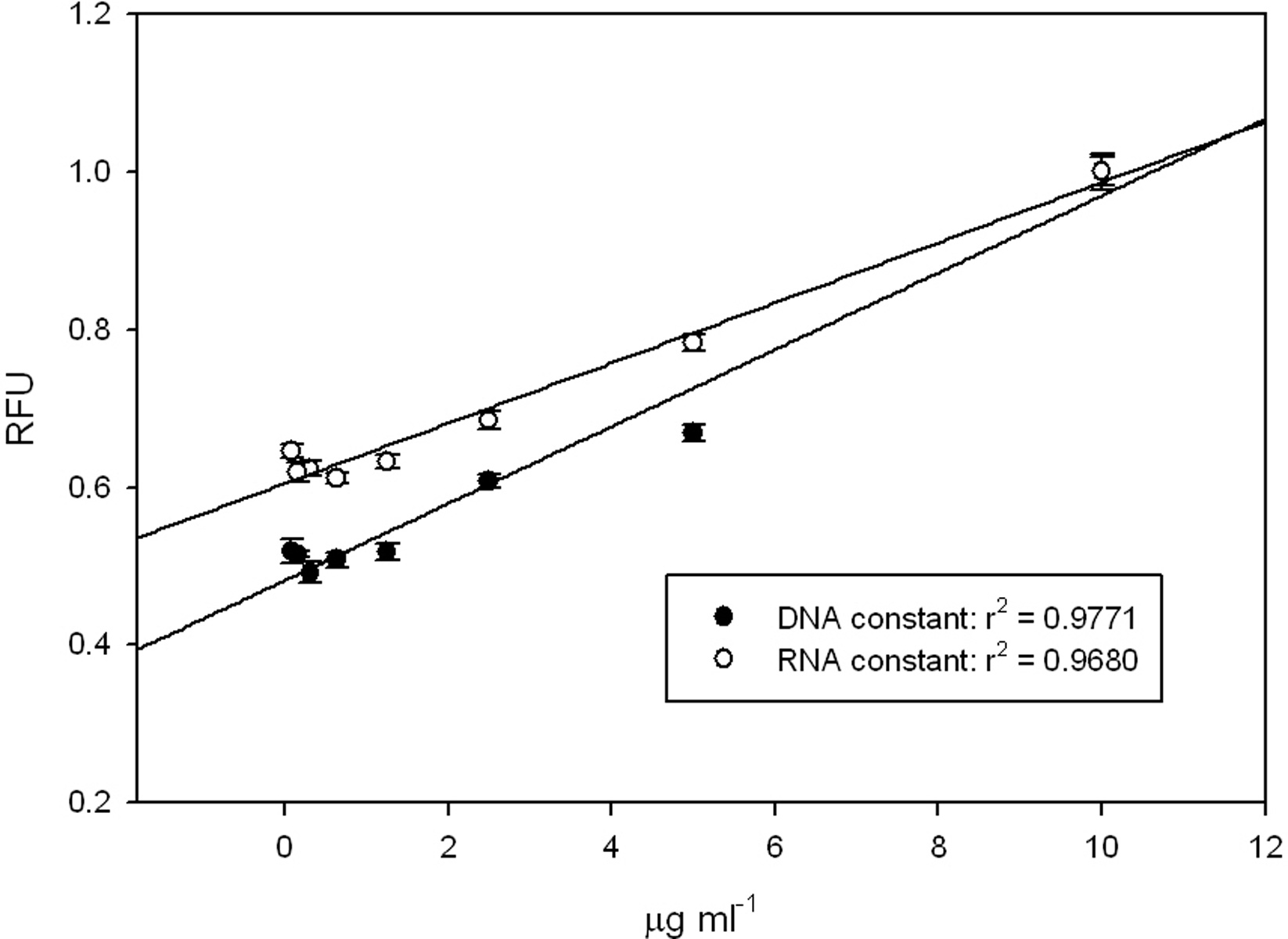

Supplement: Additional file 1 — DNA: RNA binding assay. Graph represents 132A staining of RNA and DNA in a mixed population, with one nucleic acid kept at a steady state and the other serially diluted, where the maximum fluorescence intensity was set to one. Steady state RNA was observed to have a higher fluorescence intensity than serially diluted RNA, indicating 132A has a higher fluorescent signal when bound to RNA. [file 1471-2121-10-45-S1.jpeg]

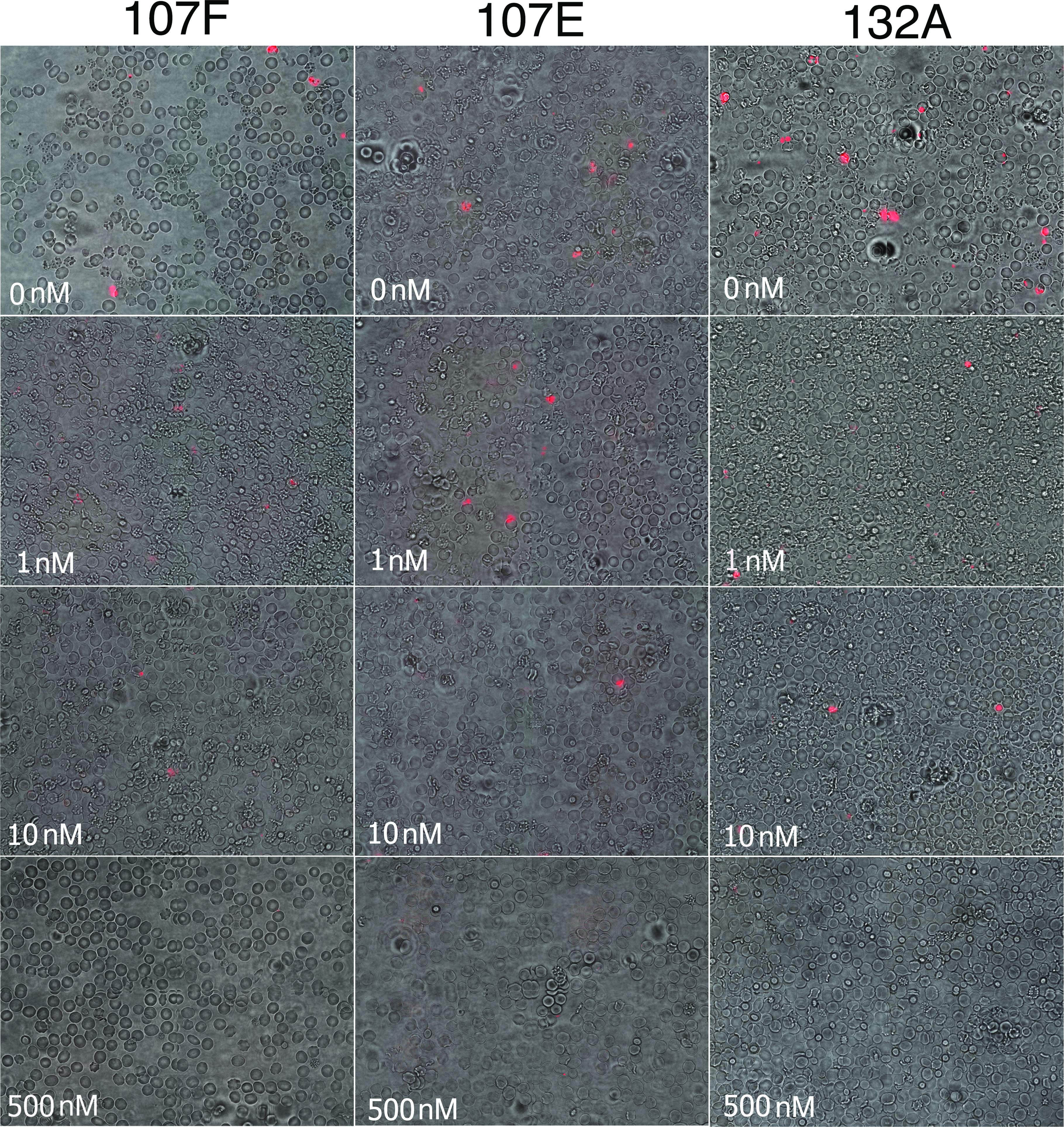

Supplement: Additional file 2 — Merged montage images. A tiling of two by two images of transmitted light and fluorescent parasites, were taken with the Pathway HT. Decreased parasitemia with increasing concentration of chloroquine was observed with the 3D7 strain. [file 1471-2121-10-45-S2.jpeg]
